# Geographical survey of the intermediate host of *Schistosoma japonicum*: Toward precise management of *Oncomelania hupensis*

**DOI:** 10.1371/journal.pntd.0008674

**Published:** 2020-10-07

**Authors:** Xiaowei Shan, Si Liu, Jianbing Liu, Hong Zhu, Ying Xiao, Yanyan Chen

**Affiliations:** Hubei Provincial Center for Disease Control and Prevention, Wuhan, China; University of Florida, UNITED STATES

## Abstract

Schistosomiasis caused by *Schistosoma japonicum* is a public health concern in China, and Hubei is one of the most affected provinces. Although the routine surveillance since the mid 1950s has generated substantial data pertaining to the habitats of the intermediate snail host, *Oncomelania hupensis*, its spatiotemporal distribution is not known. A review of local chronicles on the annual records of schistosomiasis control program was conducted to retrospectively collect information about *O*. *hupensis* habitats. The habitats were mapped by a field survey in 2016. We categorized the habitats into five evolutionary types, namely, Type I, current habitat; Types II-IV, historical habitat; and Type V, suspected habitat according to habitat development. The shape of habitats was determined using geographical information systems. A visual database was established and managed on the ArcGIS platform. A total of 43 472 *O*. *hupensis* habitats, covering an area of approximately 430 000 hectares, were identified through the study. Over 60% of these habitats have been eliminated. The highest number of *O*. *hupensis* habitats was recorded in 1975; however, most of them were preserved until 1995. Our study, for the first time, sheds light on the spatiotemporal distribution of *O*. *hupensis* in the most affected province in China. The data will be valuable for policy making and for formulating strategies to eliminate schistosomiasis in Hubei Province.

## Author summary

Schistosomiasis is a major infectious disease of humans and animals, and is a global concern. In China, it is endemically found across 12 provinces along the Yangtze River and in the area south of the river. According to the “13th Five-Year Plan for National Schistosomiasis Control”, by the end of 2020, schistosomiasis epidemic will continue to decline towards eradication throughout China. However, the control and elimination of its intermediate snail host, *Oncomelania hupensis*, is crucial for blocking schistosomiasis transmissions in China. Therefore, further understanding, surveillance, and control of *O*. *hupensis* distribution is critical to protect the health of individuals in endemic areas and to formulate relevant prevention and control strategies in order to reduce the burden of schistosomiasis, and ultimately overcome this epidemic. In this study, we reviewed local chronicles and annual records and extensively surveyed Hubei Province to collect information on the historical, current, and suspected habitats of *O*. *hupensis*. Subsequently, we used various geospatial tools (ArcGIS and Google Earth) to visualize the spatial distributions of habitats. Our study provides valuable data that will facilitate more effective habitat management of this intermediate host species and contribute to more precise management of schistosomiasis in China.

## Introduction

Schistosomiasis is a global infectious disease caused by several parasitic flatworms belonging to the genus *Schistosoma*. This infectious disease has been reported in 78 countries, endangering human health and depressing social and economic development worldwide. Over the past few decades, approximately 230 million people have been infected with *Schistosoma* and nearly 800 million individuals have been threatened with the infection [1–3].

Schistosomiasis is a major concern in the People's Republic of China in terms of economic development and public health [4,5], as it is endemic to 12 provinces located along the entire Yangtze River, and in the area south of the river. Considerable achievements in schistosomiasis control are attributable to national control programs initiated 60 years ago in China [6,7]. Schistosomiasis transmission has also been significantly reduced since 2004, following the implementation of a new nationwide control strategy, which focused on controlling the source of infection (i.e. livestock management), rather than mass human chemotherapy. This strategy has facilitated progression toward schistosomiasis elimination in China [8,9]. For example, at the end of 2016, five provinces (Shanghai, Zhejiang, Fujian, Guangdong, and Guangxi) achieved the standard of schistosomiasis elimination and the remaining seven provinces (Sichuan, Yunnan, Jiangsu, Hubei, Anhui, Jiangxi, and Hunan) achieved the standard of schistosomiasis transmission control [10,11].

Hubei Province, located in the middle reaches of the Yangtze River, was once one of the most severely affected endemic areas. Schistosomiasis is endemic to 63 counties, accounting for 62% of the counties in the province [12]. Hubei Province successfully achieved the national standard of schistosomiasis morbidity control in 2008 and further achieved the national standard of schistosomiasis transmission control in 2013 [13]. Although disease prevalence in most counties declined to zero, risk factors associated with schistosomiasis transmission persist in local areas.

The blood fluke, *Schistosoma japonicum*, causes schistosomiasis japonica (intestinal schistosomiasis). *Oncomelania hupensis* is a small tropical freshwater snail (1 cm long and 0.4 cm wide, generally) that is a unique intermediate host of *S*. *japonicum* [14]. Furthermore, *S*. *japonica* transmission significantly depends on the distribution of this host species [15]. Therefore, understanding the distribution of *O*. *hupensis* facilitates the determination of areas susceptible to schistosomiasis japonica and allows effective control measures. In accordance with the schistosomiasis control programs initiated in the mid 1956s, the surveillance and control of snail intermediate hosts were systematically conducted by annually recording their distribution and mapping their habitats. Therefore, to assess *O*. *hupensis* distribution and infer schistosomiasis japonica risk in Hubei Province, we conducted an ecological survey to 1) establish a visual database of *O*. *hupensis* habitats in this region and 2) further characterize these habitats according to various parameters.

## Methods

We conducted a local chronicle reviews and annual records of prevention and control work reviews and carried out a field surveys in Hubei Province in 2016. We retrospectively collected relevant information on all types (including historical, current, and suspected) of *O*. *hupensis* habitats, in terms of landscape, habitat type, vegetation type, and year in which the habitats were first recorded and subsequently eliminated within the region. A field survey was conducted on all types of *O*. *hupensis* habitats to understand their current distributions.

The collected data were cleaned to remove duplicate information, correct errors (such as invalid format), and ensure data consistency (uniform format by field qualification). Spatial data visualization was performed using ArcGIS to visualize the spatial distribution of *O*. *hupensis* habitats.

### Study area selection and *O*. *hupensis* habitat classification

Through extensive review of the local chronicles and annual records (with historical habitat information) from 1956 to 2016, we recorded *O*. *hupensis* habitats in Hubei Province and selected them for our survey. In general, *O*. *hupensis* habitat refers to a relatively independent geographical area with artificial transformations or natural formations of *O*. *hupensis* breeding conditions, which could be a man-made drainage ditch or a large natural meadow in the lake district, inclusive of various habitat types, including ditches, small reservoirs, paddy fields, dry lands, and marshlands. We divided *O*. *hupensis* habitats into three categories (current, historical, and suspected habitats) per the national industry standards [16]. Current habitat was defined as a place where *O*. *hupensis* was found within two consecutive years. Historical habitat was defined as a place where *O*. *hupensis* was eliminated for over 2 years. Suspected habitat was defined as a place adjacent to the current habitat but without *O*. *hupensis*. Each habitat was identified by a unique 13-digit code for convenient management.

Based on these three categories, all habitats were further categorized into five evolutionary types. In other words, the change and development of an *O*. *hupensis* habitat, for example, a habitat that was a ditch historically, but with land management, has been changed to a paddy field now. Type I, current habitats, where the breeding conditions of *O*. *hupensis* are still available; Type II, historical habitats exhibiting intact physical and ecological conditions, and thus, are suitable for *O*. *hupensis* re-emergence; Type III, historical habitats exhibiting slightly altered physical and ecological conditions; Type IV, destroyed historical habitats that are no longer suitable for *O*. *hupensis* breeding; and Type V, suspected habitats adjacent to known habitats and therefore likely suitable, but in which *O*. *hupensis* has never been recorded. Among them, Type I represents current *O*. *hupensis* habitats, Type II-IV represent historical *O*. *hupensis* habitats, and Type V represents suspected *O*. *hupensis* habitats. To distinguish among Types II-IV, we set an indicator to quantify “intact” “slightly” and “destroyed” historical habitats. Type II is the “intact” historical habitat, where 100% of the area is still suitable for *O*. *hupensis* breeding. Type IV is the “destroyed” historical habitat that has changed entirely through measures such as land hardening and completely unsuitable for *O*. *hupensis* breeding. Type III is the “slightly” historical habitat, where the ratio of habitat change is between 0 and 100; for example, if 60% of a habitat has changed, it was classified as Type III.

Over the years, *O*. *hupensis* habitats have evolved due to various anthropogenic activities (e.g., cementing riverbanks, urbanization, engineering construction, water storage for fish, and shelter forest projects). Consequently, we aggregated the different *O*. *hupensis* habitats (e.g., ditches and paddy field) in the historical and current habitat types by evolutionary types to examine the overall evolution of these habitats as statistics in radar graph.

### Field survey of *O*. *hupensis* historical, current, and suspected habitats

In 2016, a field survey was carried out in all 5388 schistosomiasis endemic villages in 514 townships in 63 counties in 13 cities of Hubei Province, listed in the 2016 annual report on prevention and control of schistosomiasis. Historical and suspected habitats were investigated using an environmental sampling method [16] to search for *O*. *hupensis* in suspected vegetation. A systematic sampling method [16] with 0.1 m^2^ quadrats placed 5 or 10 m apart was used for further investigation, if *O*. *hupensis* was found in the first step. For the current habitats, the systematic sampling method was employed. All collected *O*. *hupensis* individuals were transported to the lab, where they were examined for *Schistosoma* cercariae [17]. After removing their shells, the *O*. *hupensis* were mounted on glass slides and analyzed using a dissecting microscope.

### Digitalization of habitats

In the field survey, control-point coordinates of each habitat were obtained using a Global Positioning System (GPS, WGS84). For linear habitats (e.g. ditches), two coordinates at the initial and terminal points were collected. For more complex habitats (e.g., marshlands), we acquired at least four coordinates around the habitat. We subsequently imported the coordinates obtained into Google Earth (version 7.1) and drew paths and polygons to delineate habitat shapes according to the remote-sensing images obtained from Google Earth. The historical, current, and suspected habitats were color-coded green, yellow, and brown, respectively. Finally, habitat information obtained from the local chronicle reviews and annual records of prevention and control work reviews and field surveys (e.g., landscape, habitat type, vegetation type, and year) were added as shape layer properties.

### Data analysis

ArcGIS (version 10.1) was used to visualize the spatial distribution of *O*. *hupensis* habitats. We further extracted *O*. *hupensis* habitat elevation data from the spatial information using the elevation extraction tool in Google Earth. By analyzing the mean and extreme values of *O*. *hupensis* habitat elevation, the elevation range of *O*. *hupensis* distribution was determined using SPSS (version 19.0). Radar graph and bar charts were constructed using Microsoft Excel (the 2013 edition). In the radar graph, we compared the historical and current types of each habitat, to understand the evolution of *O*. *hupensis* habitats.

## Results

### Spatial distribution of *O*. *hupensis* habitats

**Fig 1:**
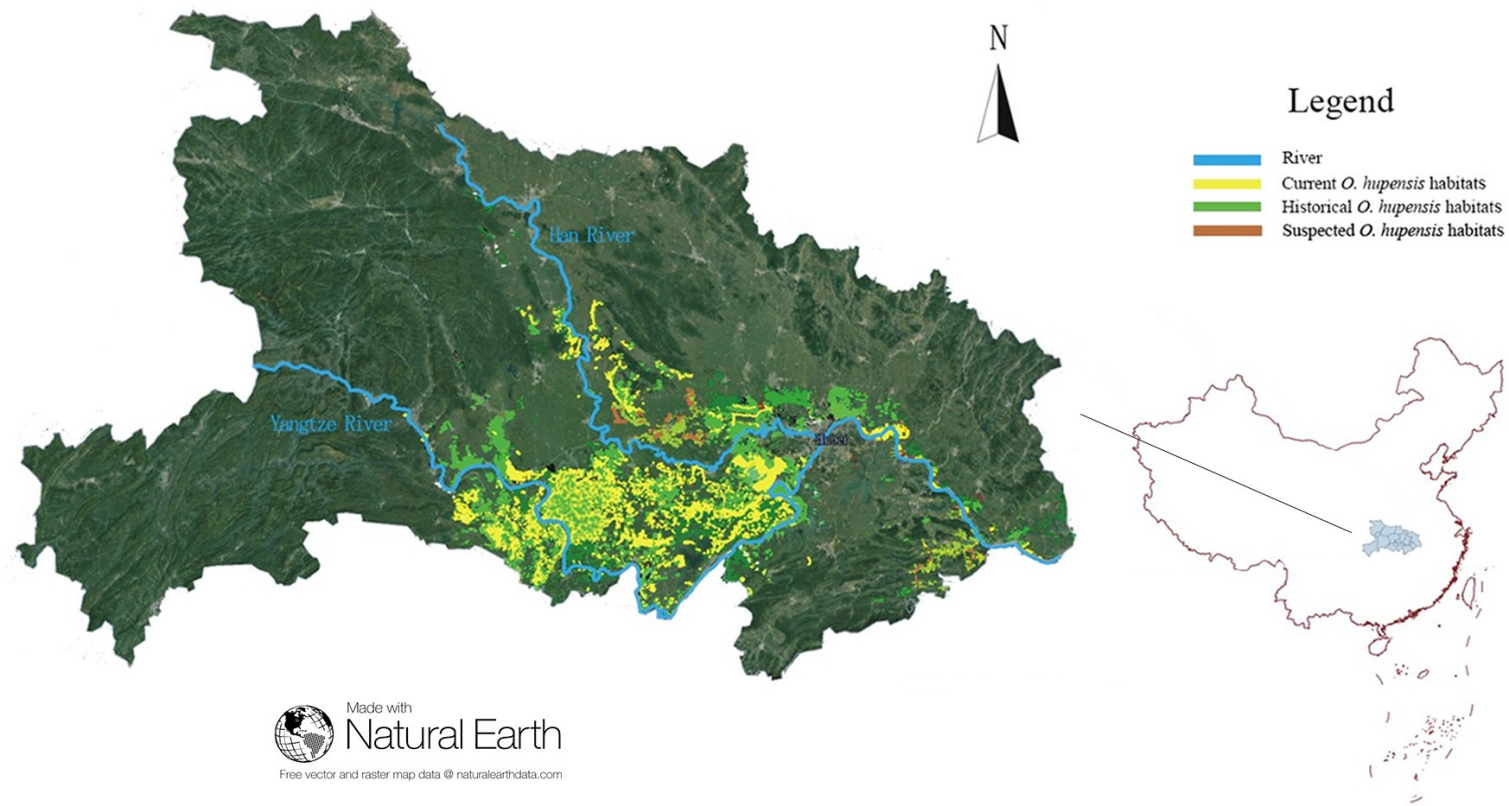
Distribution of all investigated *O*. *hupensis* habitats in Hubei Province in 2016. Hubei Province comprises the Yangtze and Han Rivers (blue area). Yellow, green, and brown indicate current, historical, and suspected habitats, respectively.

We surveyed 43 472 *O*. *hupensis* habitats that were defined as current, historical, or suspected habitats along the Yangtze and Han Rivers in Hubei Province (Fig 1). The habitat was distributed from 29°30'N to 32°07' N and from 111°13' E to 116°07' E, covering a total area of approximately 430 000 hectares. The number of quadrats in the systematic sampling survey was 4 420 342, amount of captured *O*. *hupensis* was 2 259 207, amount of live *O*. *hupensis* was 2 205 428, and average density of live *O*. *hupensis* was 0.499/0.1 m^2^. The number of quadrats in the environmental sampling survey was 4 549 077, including 11 625 captured *O*. *hupensis* and 9 418 live *O*. *hupensis*. All *O*. *hupensis* individuals collected were negative for *Schistosoma* cercariae.

**Fig 2:**
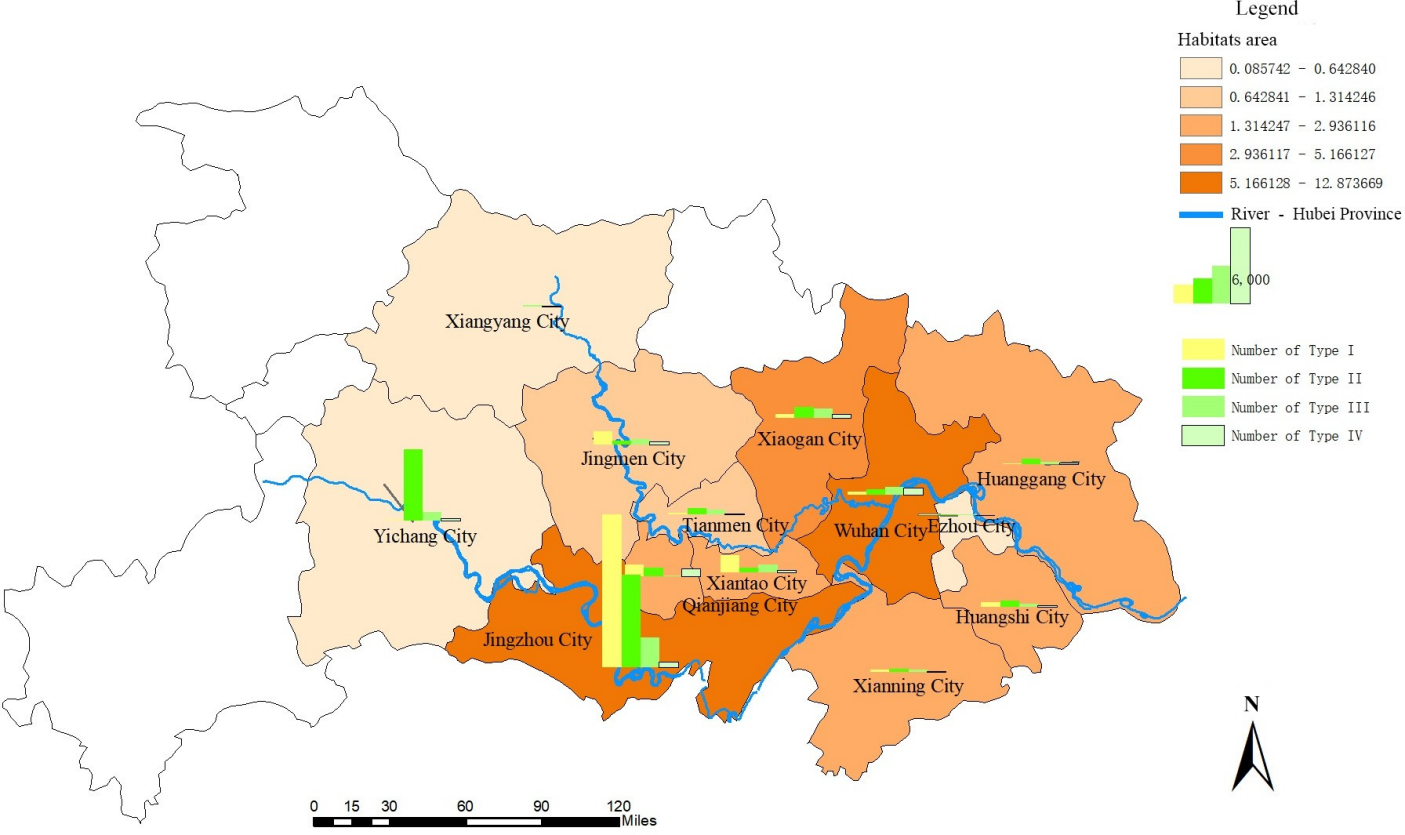
Distribution of different *O*. *hupensis* habitat types in Hubei Province. The number of *O*. *hupensis* habitats varied in the 13 cities of Hubei Province where schistosomiasis is endemic. The histogram bars represent the number of current (yellow) and historical (different greens) *O*. *hupensis* habitats.

Jingzhou City had the highest number of *O*. *hupensis* habitats (22 164; 50.98%), followed by Yichang City (6453; 14.84%) and Xiantao City (2540; 5.84%) (Fig 2). Specifically, there were 16 718 current (Type I) *O*. *hupensis* habitats, accounting for 38.45% of the total habitats in Hubei Province. Among these, 12 079 (72.25%) were in Jingzhou City, 1366 (8.17%) were in Xiantao City, and 1024 (6.13%) were in Jingmen City (Fig 2). There were 26 704 (61.43%) historical *O*. *hupensis* habitats in the study area (Fig 2). Of these, 17 246 habitats (64.58%) were Type II habitats, 6473 (24.24%) were Type III, and 2985 (11.88%) were Type IV, indicating that approximately 88.82% of all historical *O*. *hupensis* habitats were still suitable for *O*. *hupensis* breeding. There were 50 suspected (Type V) *O*. *hupensis* habitats, accounting for 0.12% of all the habitats surveyed.

### Evolution of *O*. *hupensis* habitats

**Fig 3:**
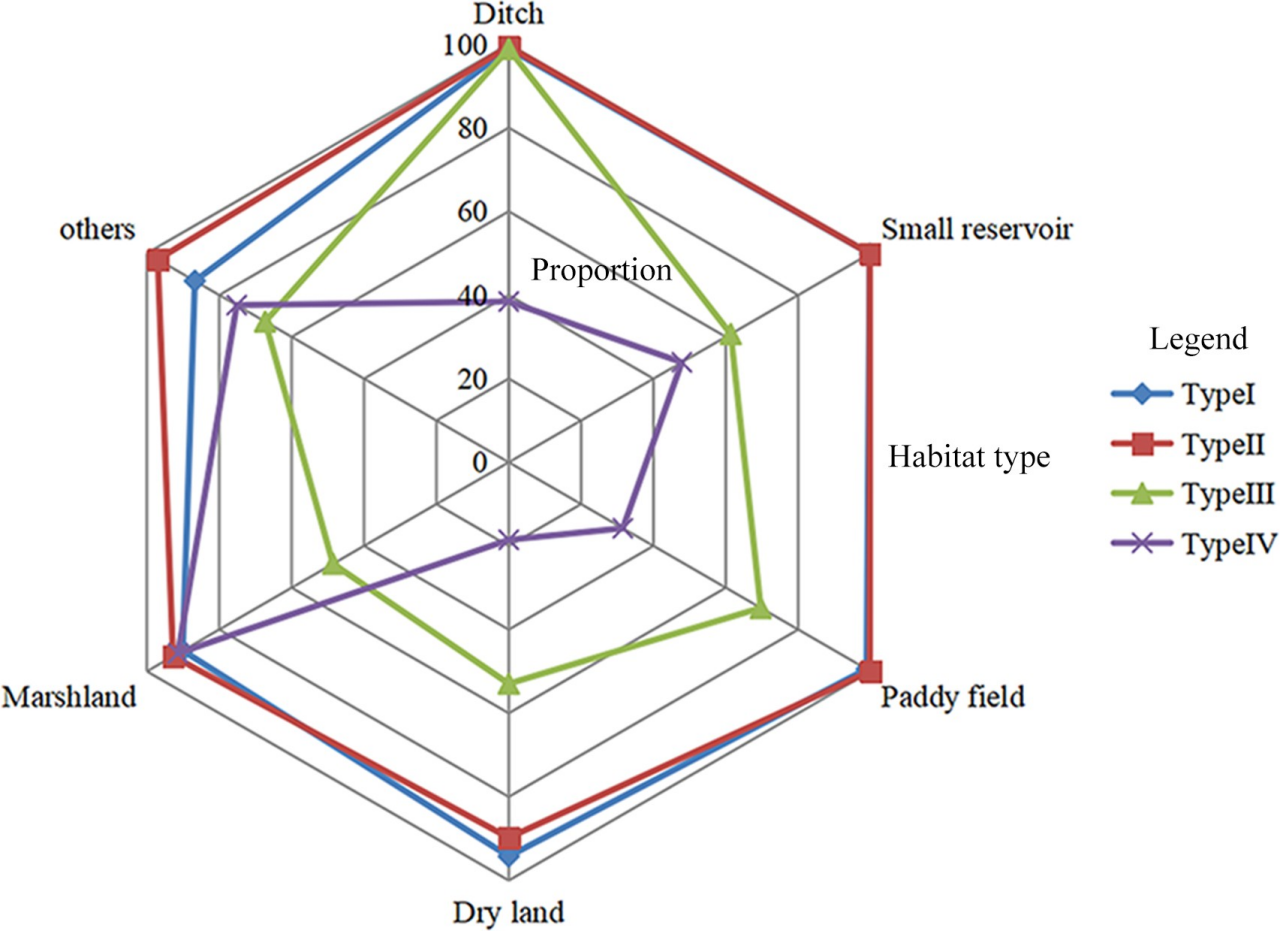
Evolution of *O*. *hupensis* habitat types in Hubei Province. The radar graph depicts changes in evolutionary habitat types. Ratios closer to 0% indicate habitat change, whereas those closer to 100% indicate a lack of change.

Through the radar graph analysis, the proportion of Type I and II habitats that did not change was similar (Fig 3). Irrespective of Type I or II habitat, the marshland and ditch habitats changed marginally, despite being affected by engineering construction (Fig 3). Although the transformation of dry land, small reservoir, and paddy field habitats was distinct, they were easier to eliminate given their evolutionary type (Type I or II) (Fig 3). Type V habitats showed no changes and were not shown in the radar graph.

**Fig 4:**
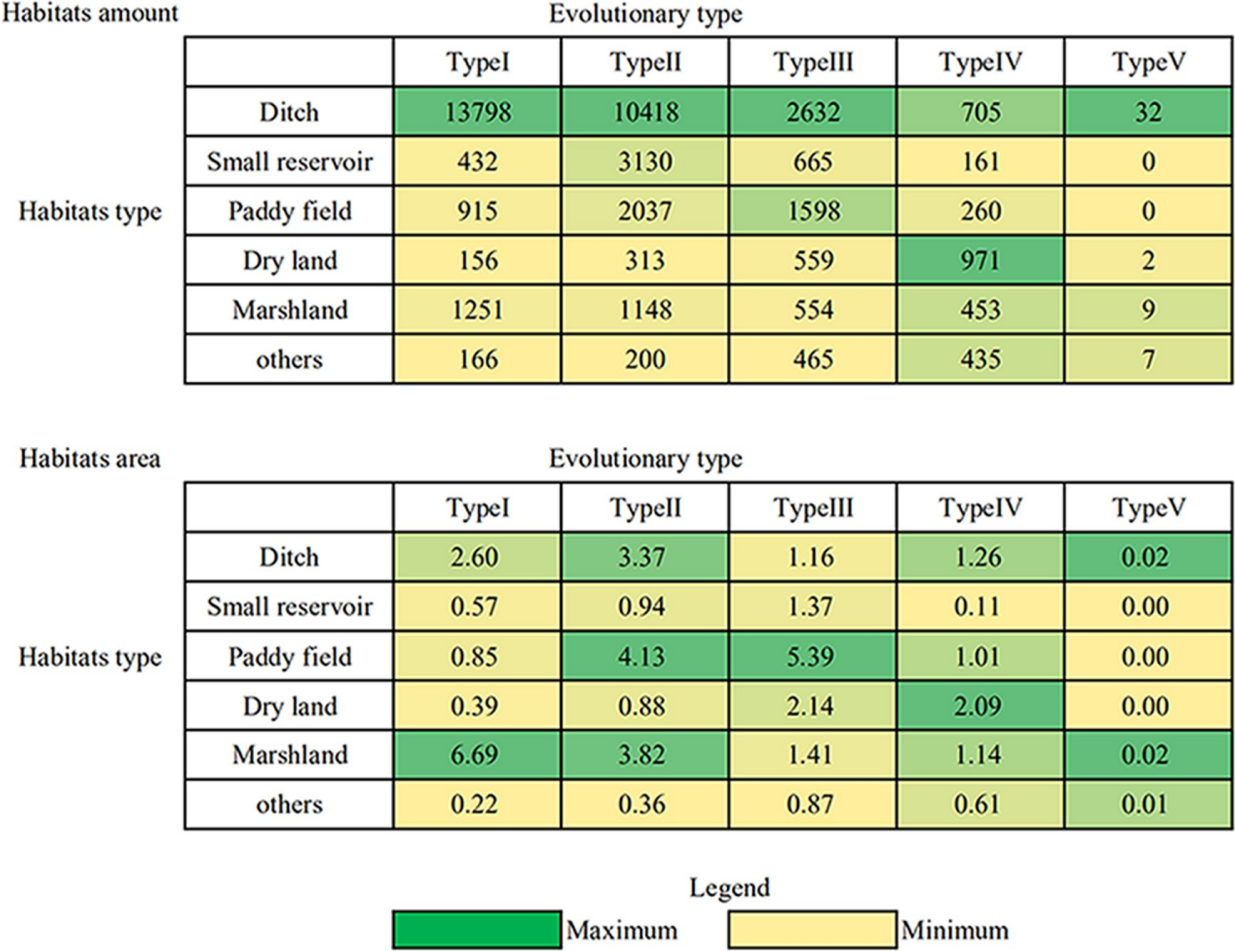
Proportion of *O*. *hupensis* habitat number and habitat area in different evolutionary types in Hubei Province. The green-yellow scale is adopted, in which green represents the maximum value and yellow represents the minimum value. Ditch habitats were categorized as Type I and were the most numerous, whereas Type I marshlands had the highest habitat area.

Among Type I habitats, ditch habitats were the most numerous, comprising 82.53% (13 798) of the sampled Type I *O*. *hupensis* habitats, followed by the marshlands (7.48%; 1251) (Fig 4). Alternatively, the marshlands comprised the largest area of Type I habitats, accounting for 59.10% of the sampled area in this evolutionary habitat type (Fig 4). Among Type II habitats, the ditch habitats comprised 60.41% (10 418) of the sampled habitats, indicating that *O*. *hupensis* ditch habitats were widely distributed, but the associated area was smaller and thus these habitats were easier to eliminate.

### Temporal discovery and elimination of *O*. *hupensis* habitats from 1956 to 2016

**Fig 5:**
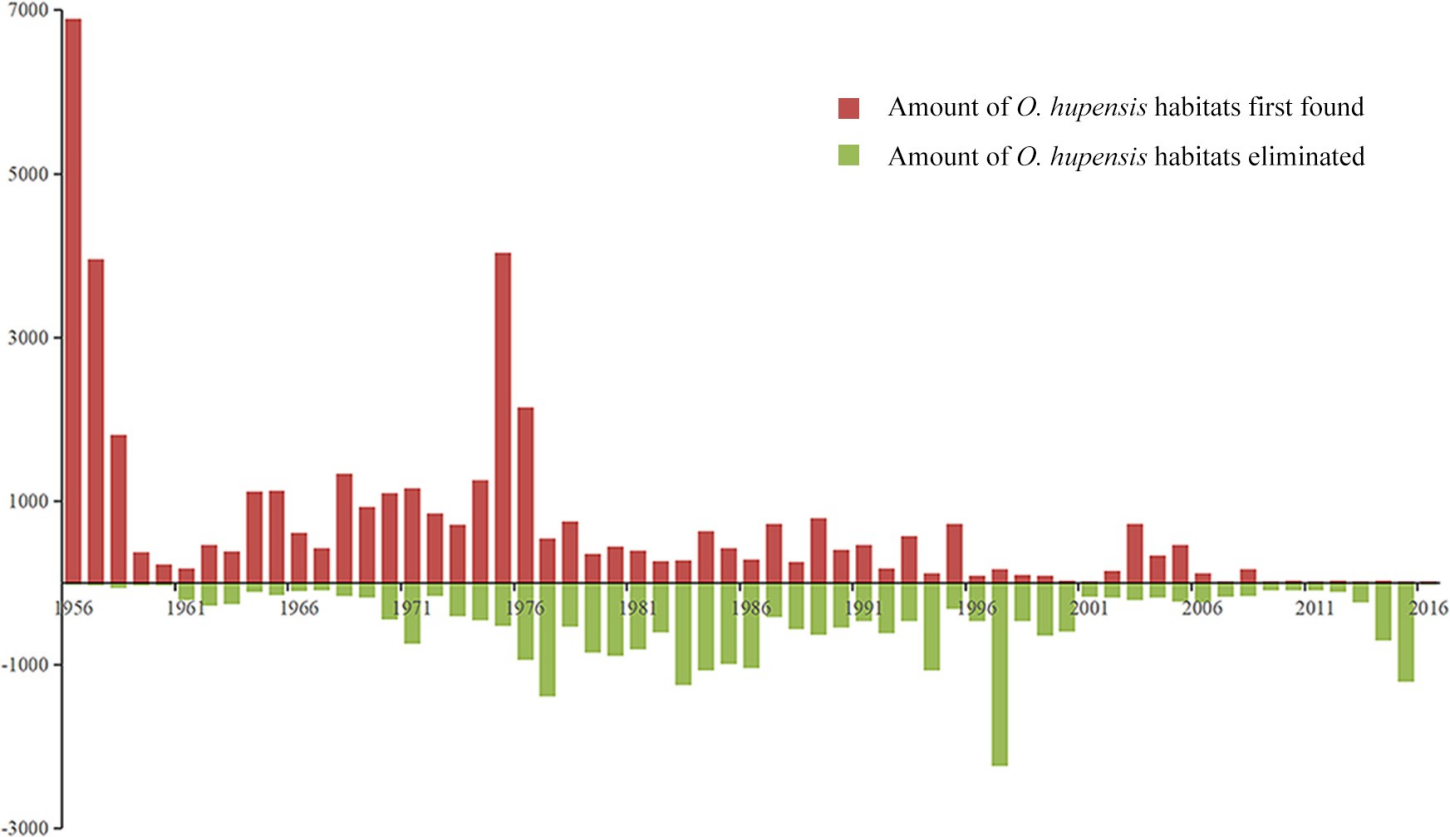
Annual discovery and elimination of *O*. *hupensis* habitats in Hubei Province.

**Fig 6:**
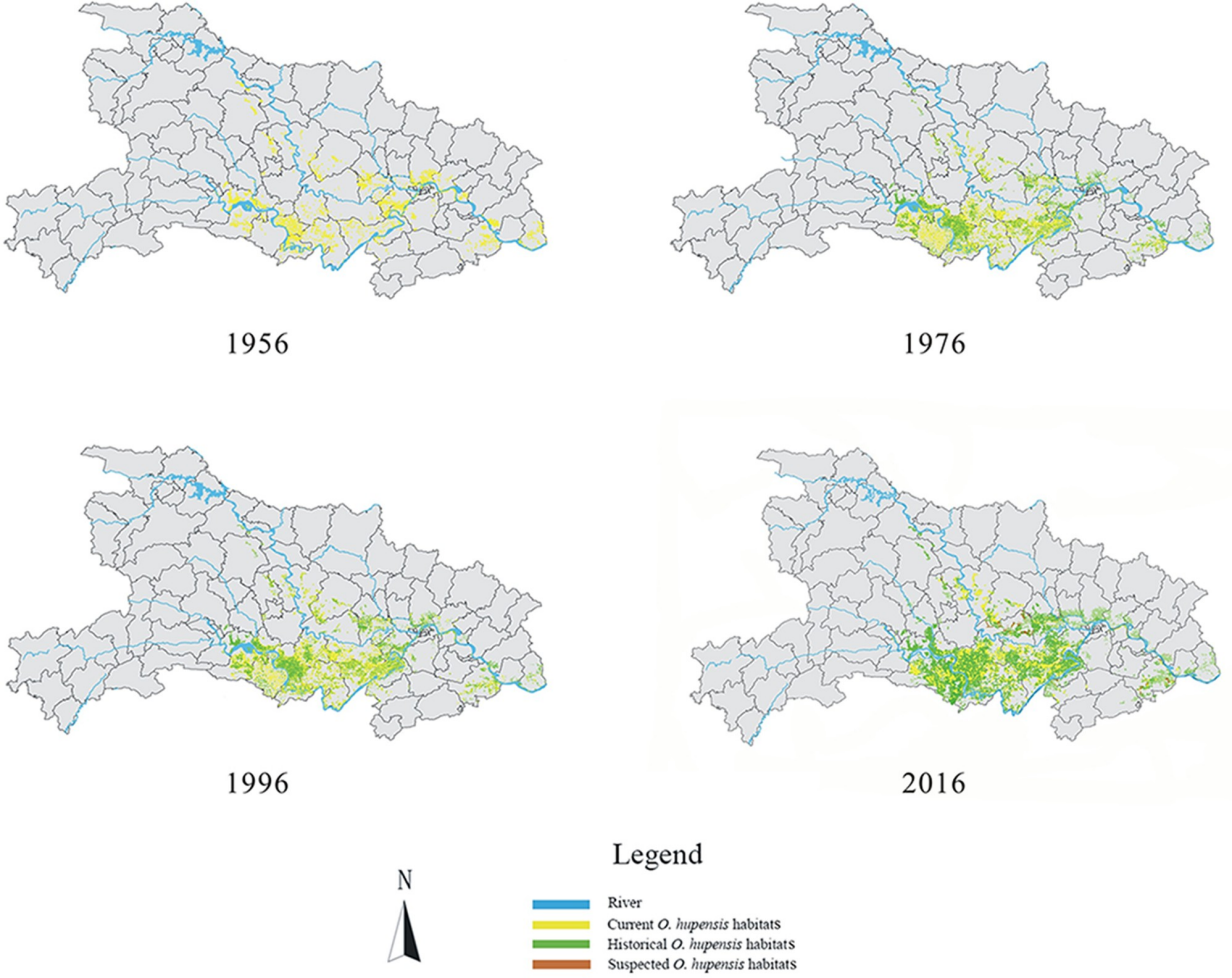
Distribution of *O*. *hupensis* habitats in four key time nodes from 1956 to 2016 in Hubei Province. Four key time nodes 1956, 1976, 1996, and 2016 were chosen to display the evolution of *O*. *hupensis* habitats. With time, more *O*. *hupensis* habitats were found, and more *O*. *hupensis* habitats have been eliminated in Hubei Province.

*Oncomelania hupensis* habitats were first recorded in Hubei Province in 1956, whereas most recently, habitats were observed in 2016. The highest number of *O*. *hupensis* habitats were recorded in 1975, and most of these habitats were subsequently eliminated 20 years later (1995) (Fig 5). We chose four key time nodes from 1956 to 2016 (1956, 1976, 1996, and 2016) to display the evolution of *O*. *hupensis* habitats in Hubei Province (Fig 6). With time, the number of *O*. *hupensis* habitats increased and more *O*. *hupensis* habitats have been eliminated because of the implementation of prevention and control work of schistosomiasis in Hubei Province.

### Comparative analysis of elevation of different *O*. *hupensis* habitat types

We extracted elevation data of 43 472 *O*. *hupensis* habitats from the Google Earth topographic map using the elevation extraction plug-in. A total of 39 093 elevation data were included in the analysis after removing 4379 data. The highest elevation of the current *O*. *hupensis* habitats was 303.77 m, whereas the lowest elevation was 9.01 m, with a median elevation of 29.32 m. Regarding the historical *O*. *hupensis* habitats, the highest, lowest, and median elevations were 330.23, 9.01, and 30.49 m, respectively. The difference between the median elevation of current and historical habitats was 1.17 m. Wilcoxon test showed that the difference was significant (*P* < 0.05).

## Discussion

In China, prevalence of schistosomiasis japonica is affected by not only the distribution of its intermediate host, *O*. *hupensis*, but also its associated socioeconomic and environmental factors. Nonetheless, over the years, China has been successful in controlling the transmission of this infectious disease. According to the “13th Five-Year Plan for National Schistosomiasis Control,” [10] by the end of 2020, Sichuan, Jiangsu, Yunnan, Hunan, and Hubei Provinces will realize the transmission-blocking standard, and over 90% and 70% of counties in Anhui and Jiangxi Provinces where schistosomiasis is endemic, respectively, will achieve the transmission blocking standard [10].

Despite these achievements, there are many challenges in achieving the goals of the “13th Five-Year Plan for National Schistosomiasis Control,” especially regarding the investigation and control of *O*. *hupensis*, given that the necessary techniques should be further improved. Additionally, some *O*. *hupensis* habitats cannot be effectively controlled, because in doing so, aquaculture areas could be severely affected owing to the negative effects of molluscacides on fish. Furthermore, excavation of the Yangtze River by water conservation projects will likely create new *O*. *hupensis* habitats as beach areas, which will be consequently expanded [18]. Given that the control and elimination of *O*. *hupensis* are key aspects for successful blocking of schistosomiasis transmission in China, it is critical to further elucidate the distribution of this intermediate host species, to ultimately formulate relevant prevention and control strategies and thus protect human health in schistosomiasis-endemic areas [19]. Thus far, the effective approaches have been the use of molluscacides and implementation of environmental modifications to reduce *O*. *hupensis* density and suitable habitat areas, respectively [20,21].

In the present study, we revealed that since 2006, the integrated prevention and control strategies in Hubei Province have successfully controlled *O*. *hupensis*. The spread of *Schistosoma japonicum* to *O*. *hupensis* habitats has reduced due to the replacement of cattle with machines, improvement in drinking water and lavatories, elimination of cattle in counties where the disease was prevalent, prohibition of cattle grazing in *O*. *hupensis* habitats, and other agricultural measures. In fact, from 2012 to 2018, schistosomiasis-positive *O*. *hupensis* was not found, even in the present study; nonetheless, *O*. *hupensis* habitats have not been effectively reduced in Hubei Province. Thus, there is a risk of schistosomiasis transmission in this area due to the existence of multiple *O*. *hupensis* habitats and thus widespread distribution of this species, as indicated in the present study.

The results of our field surveys on *O*. *hupensis* distribution in Hubei Province indicated that *O*. *hupensis* habitats including the current and historical habitats mainly comprised ditch-type habitats. This is particularly concerning because ditches are utilized as irrigation and drainage channels and thus produce domestic water supplies. Therefore, it is critical to eliminate this species from these habitats to reduce the risk of *O*. *hupensis* diffusion throughout aquatic habitats [22]. Furthermore, this result suggests that *O*. *hupensis* elimination in ditch habitats is a key strategy for overall control of this intermediate host species in Hubei Province, and therefore, quality control should be strengthened and continuously conducted in these habitats.

We further revealed that Hubei Province mainly comprised Type I (current) and Type II (in-tact historical) *O*. *hupensis* habitats. Additionally, Type I habitats were target for the prevention and control of schistosomiasis, whereas Type II habitats were targets for the surveillance of schistosomiasis. We also revealed that the change in ditch and marshland habitats did not play a major role in the control of *O*. *hupensis*. On the contrary, the documented changes in dry land, small reservoirs, and paddy field habitats were more likely to facilitate successful *O*. *hupensis* elimination. Currently, only 6.87% (2985) of all surveyed *O*. *hupensis* habitats have completely changed. In addition to our results, it is important to consider that Hubei Province is in the middle reaches of the Yangtze River, and therefore, *O*. *hupensis* individuals are easily transferred by floods. Thus, despite achieving the elimination standard of schistosomiasis transmission in 2018, it is critical to continuously monitor the historical and current *O*. *hupensis* habitats to ensure that the standard is maintained in this region [23].

The peak appeared in 1975, and this is associated with the political background of schistosomiasis mass prevention and treatment programs. The Chinese Government has prioritized schistosomiasis control. This work has been conducted in several stages, and from 1955 to 1976, the mass prevention and treatment process was conducted. This stage was mainly characterized by combining the characteristics of the rural collective economic development with large-scale mass prevention and control measures, and this process plays an active role in controlling *O*. *hupensis*. During this period, we discovered many new *O*. *hupensis* breeding habitats, resulting in the peak that appeared in Hubei Province [24]. With time, more *O*. *hupensis* habitats were found; furthermore, more *O*. *hupensis* habitats have been eliminated because of the implementation of prevention and control measures of schistosomiasis.

Rapid economic and societal development has resulted in the construction of aquaculture and wetland areas, which increase the input of aquatic animals and plants into aquatic areas. Therefore, in the future, further control of *O*. *hupensis* in marshlands along the Yangtze River will be crucial, and more focus should be on determining the most effective and safe ecological methods of control in these areas. The median elevation of the current *O*. *hupensis* habitats is lower than that of the historical habitats by approximately 1 m. Historical literature revealed that the distribution of *O*. *hupensis* varies with the water flooding time at different elevations. *O*. *hupensis* is mainly distributed in water at suitable elevations for 1 to 5 months. It is distributed in the middle reaches of the Yangtze river, at the height of 20–30 m [25]. It can be concluded that habitats at higher elevations are more likely to be eliminated in Hubei Province. Thus, there is a stronger need to formulate different control strategies for habitats at lower and higher elevations in Hubei Province. Similarly, a previous study reported that following the construction of the Three Gorges Dam, the average annual water level in the middle and lower reaches of the Yangtze River decreased, and in *O*. *hupensis* habitats in the lakes and marshes [26]. Literature shows that after the construction of the Three Gorges Dam, the water level of the Yangtze river reduces by approximately 1 m during the flood period, which will be conducive to the breeding of *O*. *hupensis* and adverse to the control of *O*. *hupensis*. Therefore, it is reasonable that a difference of approximately 1 m between the elevation of the historical and current *O*. *hupensis* habitats was found in our research.

Despite the decline of schistosomiasis in Hubei Province, the demand and need for continuous disease monitoring has increased [27]. Therefore, a part of the new frontier in public health research involves the adaptation of technologically advanced and expensive concepts for managing operations in resource-poor cities, by developing low-cost tools and solutions [28]. As the social economy rapidly develops, the natural appearance of *O*. *hupensis* habitats has significantly changed. For example, the replacement of technical personnel who study and maintain *O*. *hupensis* records and the loss of historical data pertinent to the understanding of *O*. *hupensis* distribution and thus schistosomiasis transmission, will hinder continuous and effective prevention and control of this disease in Hubei Province.

To prevent this, Google Earth proved to be a useful and effective tool in the present study, given that it facilitated the identification of *O*. *hupensis* habitats, provided basic information for them, and permitted visualization of their spatial distributions. In doing so, we provide more accurate spatial information that could be used for decision making and implementing more intense and effective management of these habitats [28,29]. In the future, these map layers could be used internally to guide the prevention, surveillance, and control of schistosomiasis in high-risk areas, and could be further distributed externally to inform the public and local decision-makers of spatial risk patterns.

It is important to note that there are inaccuracies in the historical data collected for use in the present study. The data we collected dated back to 1956; since then, there have been many changes in governmental administration, and likely many undocumented changes in *O*. *hupensis* habitats. Thus, it was difficult to accurately and confidently determine the years in which *O*. *hupensis* habitats were first recorded, eliminated, and tested positive for *Schistosoma*. The density of *O*. *hupensis* varies with the changes in temperature and rainfall during different seasons. Temperature and rainfall were not investigated, which made it difficult to effectively analyze such data in the later period. This will be the direction of our future research.

## Conclusions

In this study, we accurately determined the location of *O*. *hupensis* habitats in Hubei Province using the GPS and established an accurate spatial database of their habitats in this region. The electronic map that we constructed accurately reflects the distribution of *O*. *hupensis* habitats in Hubei Province. Thus, this map can be used to effectively prioritize disease control strategies and target disease interventions to high-risk areas in order to eliminate the risk of epidemic schistosomiasis. The increased availability of these epidemiological tools will allow targeted and effective disease interventions in areas of greatest need, and thereby help conserve scarce resources and reduce the burden of schistosomiasis in China.
